# The modifying effect of the building envelope on population exposure to PM_2.5_ from outdoor sources

**DOI:** 10.1111/ina.12116

**Published:** 2014-05-10

**Authors:** J Taylor, C Shrubsole, M Davies, P Biddulph, P Das, I Hamilton, S Vardoulakis, A Mavrogianni, B Jones, E Oikonomou

**Affiliations:** 1 Bartlett School of Graduate Studies, UCLLondon, UK; 2 UCL Energy Institute, The Bartlett, UCLLondon, UK; 3 Centre for Radiation, Chemical and Environmental Hazards, Public Health EnglandOxfordshire, UK; 4 Department of Architecture and Built Environment, University of NottinghamNottingham, UK

**Keywords:** PM _2.5_, Indoor air quality, Building stock model, EnergyPlus, Geographical information systems

## Abstract

A number of studies have estimated population exposure to PM_2.5_ by examining modeled or measured outdoor PM_2.5_ levels. However, few have taken into account the mediating effects of building characteristics on the ingress of PM_2.5_ from *outdoor* sources and its impact on population exposure in the *indoor* domestic environment. This study describes how building simulation can be used to determine the indoor concentration of outdoor-sourced pollution for different housing typologies and how the results can be mapped using building stock models and Geographical Information Systems software to demonstrate the modifying effect of dwellings on occupant exposure to PM_2.5_ across London. Building archetypes broadly representative of those in the Greater London Authority were simulated for pollution infiltration using EnergyPlus. In addition, the influence of occupant behavior on indoor levels of PM_2.5_ from outdoor sources was examined using a temperature-dependent window-opening scenario. Results demonstrate a range of I/O ratios of PM_2.5_, with detached and semi-detached dwellings most vulnerable to high levels of infiltration. When the results are mapped, central London shows lower I/O ratios of PM_2.5_ compared with outer London, an apparent inversion of exposure most likely caused by the prevalence of flats rather than detached or semi-detached properties.

**Practical Implications**

Population exposure to air pollution is typically evaluated using the outdoor concentration of pollutants and does not account for the fact that people in London spend over 80% of their time indoors. In this article, building simulation is used to model the infiltration of outdoor PM_2.5_ into the domestic indoor environment for dwellings in a London building stock model, and the results mapped. The results show the variation in relative vulnerability of dwellings to pollution infiltration, as well as an estimated absolute indoor concentration across the Greater London Authority (GLA) scaled by local outdoor levels. The practical application of this work is a better understanding of the modifying effect of the building geometry and envelope design on pollution exposure, and how the London building stock may alter exposure. The results will be used to inform population exposure to PM_2.5_ in future environmental epidemiological studies.

## Introduction

Due to high volumes of traffic, a dense road network, and proximity to major traffic hubs such as Heathrow, London experiences a high level of outdoor PM_2.5_ air pollution relative to the rest of the UK. Population exposure to PM_2.5_ has been associated with negative health effects. In England, the fraction of mortality attributable to anthropogenic particulate air pollution in 2011 is estimated to be 5.36%, while in Greater London it is 7.17% (PHE, 2013). Earlier studies have estimated that in London in 2008, PM_2.5_ caused mortality equivalent to around 4000 deaths and that a permanent 1 *μ*g/m^3^ reduction in PM_2.5_ would add 400 000 years of life for the current population (Miller, 2010). Internationally, PM_2.5_ is estimated to cause about 3% of all mortality from cardiopulmonary disease, about 5% of mortality from cancer of the trachea, bronchus, and lung, and around 1% of mortality from acute respiratory infections in children under 5 years old (Cohen et al., 2005). While the total PM_2.5_ emissions in the UK are predicted to decrease by 25% by 2020 relative to 2009 levels, there is no known ‘safe’ level of PM_2.5_ and there will continue to be health risks associated with exposure (DEFRA, 2013).

A number of studies have examined the epidemiological relationship between exposure to pollution and negative health effects [for example, Atkinson et al. (2013) and Tonne and Wilkinson (2013)]. However, these studies focus on outdoor pollution concentrations and population health and do not account for pollution in the indoor environment. Individuals in developed countries spend the majority of their time indoors; a study of pollution exposure in different microenvironments in London found participants were spending 80% of their time indoors, with 48–53% of their time spent in their homes during summer and winter, respectively (Kornartit et al., 2010). Therefore, the *indoor* pollution levels have a significant influence on an individual's exposure to pollution, and a building's airtightness and the manner in which it is operated can have a major impact on pollution ingress from the outdoor environment. Epidemiological studies typically use pollution measurements from urban background monitoring stations or modeled outdoor pollutant concentrations to estimate exposure; however, this may not offer a true representation of the exposure to a population spending time largely indoors. Indeed, a study of population exposure to PM_2.5_ in different microenvironments found a good correlation between residential indoor levels and personal exposures (Lai et al., 2004).

PM_2.5_ infiltration into buildings from external sources will depend on a number of factors, including the location, height, orientation, sheltering, and permeability of the building envelope, building geometry, the ventilation systems of the building, weather and urban meteorology conditions such as urban street canyons, and building occupant practices such as window opening and heating. In addition to infiltration, concentrations of PM_2.5_ in dwellings will be affected by emissions from indoor sources such as cooking, smoking, as well as general domestic activities such as cleaning, dusting, and showering (Shrubsole et al., 2012). Removal of PM_2.5_ from indoor and outdoor sources from the indoor air can occur through exfiltration, deposition onto surfaces, and filtration using mechanical ventilation systems.

Examining the relationship between indoor and outdoor pollution levels can be performed using field measurements or through modeling approaches. A number of studies have monitored the indoor concentration of PM_2.5_ in different countries [see Chen and Zhao (2011), for a comprehensive review]. In the UK, there have been field studies measuring indoor PM_2.5_ in dwellings with roadside, urban, and rural measurements (Jones et al., 2000) and distance to major roads (Kingham et al., 2000); studies comparing indoor UK levels to other European cities (Hoek et al., 2008; Lai et al., 2006); seasonal variations in indoor PM_2.5_ exposure concentrations (Mohammadyan, 2005; Wheeler et al., 2013); short-term temporal variations associated with indoor activities (Gee et al., 2002; Wigzell et al., 2013); and in the homes of individuals with respiratory illnesses (Osman et al., 2007). A comparison between different building types by Nasir and Colbeck (2013) monitored indoor PM_2.5_ levels in three different types of dwelling and found differences between them; however, differing occupant practices make it difficult to isolate the influence of the building on indoor PM_2.5_ levels.

Modeling methods have also been used to characterize the indoor concentration of PM_2.5_. Multizone mass transport models can be used to calculate concentration levels in buildings for exposure assessments (Milner et al., 2011). Studies examining the influence of ventilation and filtration interventions (Emmerich et al., 2005) and energy efficiency interventions (Das et al., 2013) on indoor PM_2.5_ concentrations have been performed using the CONTAM modeling tool. The indoor PM_2.5_ concentration across a building stock has been modeled using CONTAM for dwellings based on Boston public housing developments (Fabian et al., 2012) and the impact of energy efficient refurbishments in London's domestic stock (Shrubsole et al., 2012).

While a number of studies have examined the relationship between indoor and outdoor air pollution levels in dwellings, there has been little research on how the relative infiltration of a geographically distributed building stock can modify pollution exposure across an urban area. Chen et al. (2012) used typical infiltration rates of dwellings in US cities to estimate indoor exposure to particulate matter in a study examining short-term mortality rates; however, this study did not examine more local variations in building types and pollution levels. Furthermore, existing infiltration modeling approaches have estimated ventilation according to a schedule of activities without coupling behavior to indoor conditions such as temperature. The research presented here examines how the characteristics and geographical distribution of residential building types in the London building stock may affect the exposure levels of dwelling occupants to PM_2.5_ from external sources. The whole-building simulation tool EnergyPlus 8.0 (US-DOE, 2013) was used to model the infiltration of PM_2.5_ into the indoor environment for dwellings broadly representative of the Greater London area (GLA) building stock. Two different scenarios were considered to demonstrate the influences of the building envelope and occupant practices: (i) pollution infiltration through cracks in the building fabric only and (ii) infiltration through cracks and temperature-dependent window opening. In both cases, trickle vents were included where appropriate, while extract fans were excluded due to their intermittent use and their assumed small contribution to time-averaged indoor concentrations of outdoor PM_2.5_. Other mechanical ventilation systems, such as mechanical ventilation heat recovery (MVHR) or air conditioning (AC), were ignored due to their rarity in the UK domestic stock. The simulation results were used to develop indoor/outdoor (I/O) ratios describing the relationship between outdoor and indoor concentration of PM_2.5_. These functions were then applied to calculate the indoor concentrations of outdoor-sourced PM_2.5_ based on mapped external concentrations, temporal variations, and geographical location of dwelling types. The results were combined with existing PM_2.5_ pollution maps to understand how dwellings may affect population exposure to particulate air pollution.

## Method

**Figure 1:**
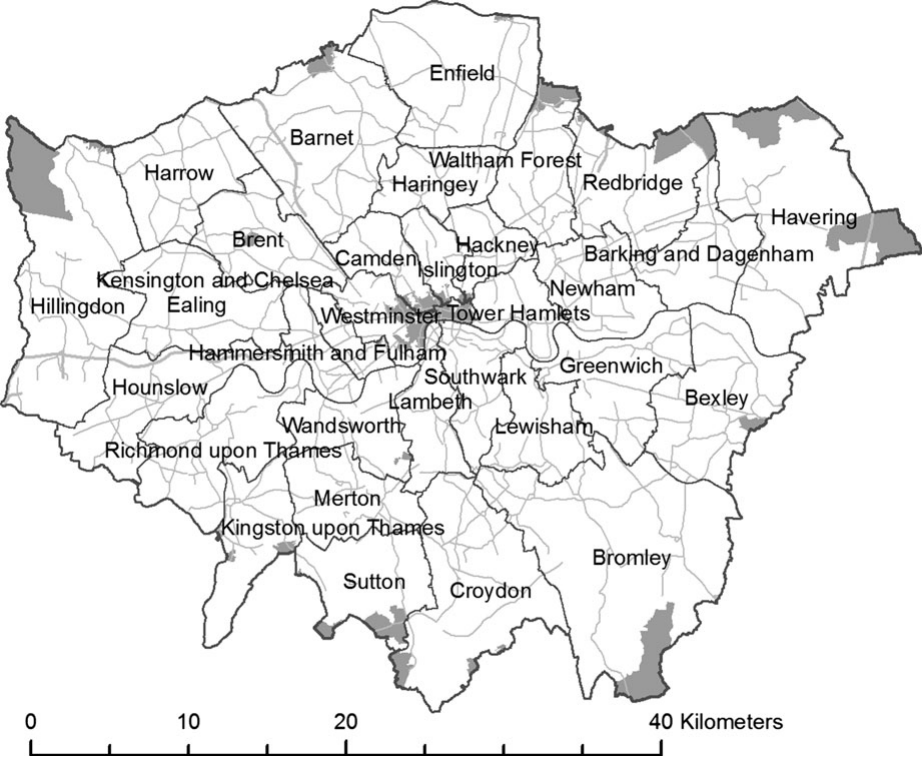
Research area: Greater London. Areas without dwelling information are shown in gray

**Figure 2:**
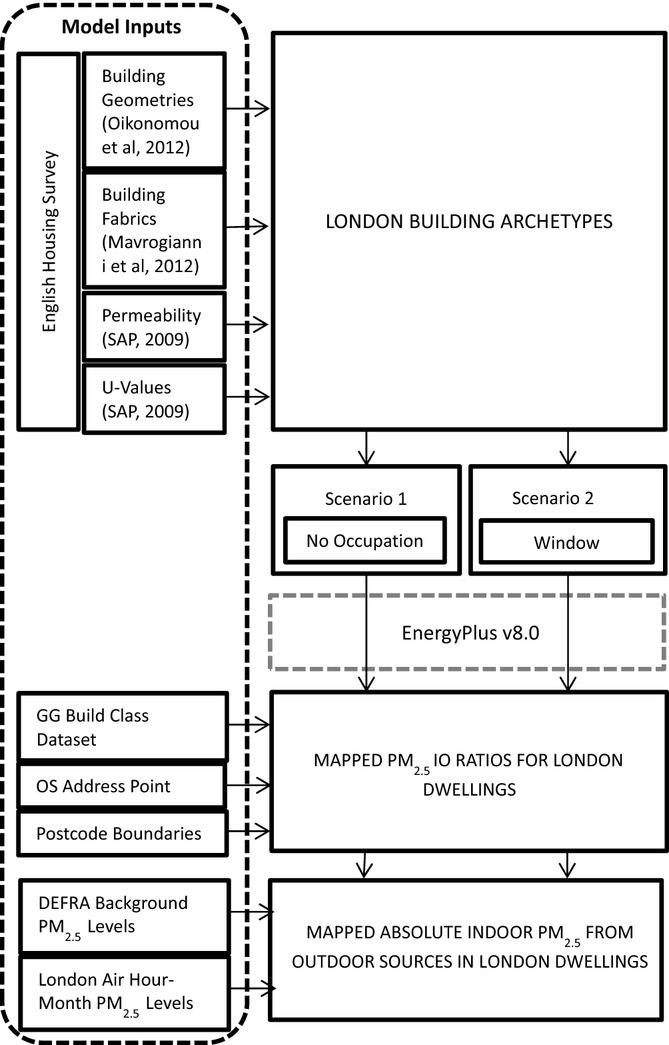
Research workflow and data inputs

The research area selected was the GLA, an area encompassing the 32 boroughs of London (Figure1). The area has good mapped coverage of building data and has been the focus of both measured and modeled studies of PM_2.5_ in outdoor air, with data on observed or estimated outdoor PM_2.5_ levels available from a range of sources. The different inputs required for the model and how they relate to the project workflow can be seen in Figure2. While the London population spends a significant amount of their time inside offices or buildings that are not their homes, commercial buildings can have significantly different indoor pollution levels due to HVAC system operation, filters, and complex building geometries. Spatial and archetype information on the commercial building stock is not widely available, and thus, this study focuses only on dwellings.

### Building archetypes

A total of 15 dwelling archetypes developed for studies into overheating risk in London were used as a basis for the EnergyPlus modeling of PM_2.5_ penetration through the building envelope (Oikonomou et al., 2012). This English Housing Survey (EHS) (DCLG, 2008), derived archetypes, with unique built form/age classifications, represents 76% of the known dwelling stock in the GLA according to the Geoinformation Group (GG) Building Class Geodatabase (GG, 2013).

Building fabrics were modeled with *U*-values derived for the building archetypes using the Standard Assessment Procedure for Energy Rating of Dwellings (SAP) (BRE, 2009), with the assumption that buildings have the most frequently occurring building fabric types according to the EHS. The building fabric can influence the indoor temperature in dwellings (Mavrogianni et al., 2012), which may lead to changes in the window opening behavior of the building occupants. Internal temperatures can also have an influence on airflow dynamics at low wind speeds due to stack effects. However, the overall effect of variations in insulation levels on yearly average indoor PM_2.5_ levels is expected to be small, and so potential retrofits were ignored. Fabric *U*-values for the building archetypes can be seen in Table S1.

The permeability of the building archetypes was determined using the methodology in the SAP documentation (BRE, 2009), which accounts for infiltration through chimneys and vents, walls, floors, and windows and increased infiltration in multistorey buildings. Infiltration rates were calculated for each building in the EHS database (with and without any reduction to the rate caused by party walls), and the mean for each built form/age classification of the archetypes in the study determined. The infiltration rates were then converted to a permeability using the ‘rule of 20’ specified in the SAP methodology. The distribution of the estimated permeabilities in the EHS was compared with that of a field measurement study (Stephen, 2000), with good results.

Simulations were run with and without the presence of trickle vents, and the results weighted according to the estimated prevalence of the vents across the UK building stock [all dwellings post-1990, and 5% of pre-1990 dwellings (DECC, 2011)], assumed to be the same as in London. A description of the building archetypes and estimated permeability can be seen in Table1.

**Table 1:** Dwelling archetype descriptions and permeability estimated from the EHS and SAP

Archetype code	Dwelling archetype	Age bracket	Frequency in stock, %	Estimated permeability (m^3^/h/m^2^ at 50 Pa)
H01	Late Victorian/Edwardian Terrace (Large T)	1902–1913	15.4	17.2
H02	WW1 & WW2 Simple Terrace	1914–1945	14.5	14.9
H03	WW1 & WW2 Large Semidetached	1914–1945	8.8	16.1
H04	‘60s & ‘70s Tall Purpose-built Flats	1960–1979	5.7	16.2
H05	Late Victorian/Edwardian Simple Terrace	1902–1913	5.5	17.2
H06	Post-War Tall Purpose-built Flats	1946–1959	4.7	13.2
H07	Recent Tall Purpose-built Flats	1980–2008	3.6	9.2
H08	Late Victorian/Edwardian Simple Terrace (attic)	1902–1913	2.9	17.2
H09	WW1 & WW2 Bungalow	1914–1945	2.4	17.9
H10	‘60s & ‘70s Simple Terrace	1960–1979	2.4	12.3
H11	‘60s & ‘70s Line-built Walk-up Flats	1960–1979	2.3	9.8
H12	WW1 & WW2 Line-built Walk-up Flats	1914–1945	2.1	10.1
H13	Recent Terrace with Shop Below	1980–2008	2.1	12.2
H14	Post-War Step-Linked Terrace	1946–1959	1.9	13.4
H15	Post-War Line-built Walk-up Flats	1946–1959	1.8	11.6

### Building simulation

Models of the building archetypes were developed in EnergyPlus, a dynamic thermal simulation tool. EnergyPlus version 8 can model airflow through buildings using the validated AirflowNetwork tool and air pollution transport using the Generic Contaminant transport algorithm. The advantage of using a coupled dynamic thermal and contaminant model is that the effect of occupant window-opening behavior in response to internal temperatures can be addressed rather than using fixed schedules. The EnergyPlus Generic Contaminant model has undergone intermodel comparison against the CONTAM model, with good results (Taylor et al., 2013).

Indoor air simulations were run for the whole year using a Prometheus Test Reference Year (TRY) hourly weather file for Islington, Central London (Eames et al., 2011), with an outdoor PM_2.5_ concentration of 14.7 *μ*g/m^3^ based on the 2010 average background concentration for the GLA (London Air, 2014). Simulations were run with four different orientations of the building (North, West, South, and East). Flats were modeled as being on a middle floor, with adjoining flats to the sides, above, and below. Dwellings with adjoining dwellings to the sides (terraced dwellings, semi-detached, and flats) were assumed to have a net air and contaminant flow of zero between the dwellings, and party walls were not modeled exposed to wind, sun, or polluted external air. Dwellings with adjoining dwellings to the top and bottom (flats) were modeled with identical dwellings above and below and shafts between levels to account for stack effects. Terraced houses were modeled as being mid-terrace with end terraces considered to be semi-detached. Indoor PM_2.5_ levels were output only for mid-floor flats as these represent the majority of dwellings in purpose-built buildings. Local wind speeds were modeled according to an urban terrain, while the solar and wind exposure effects of neighboring but unattached properties were also taken into account.

The infiltration of air was modeled through cracks in the externally exposed facades (walls, roofs, and ground floors of the buildings) and, when open, windows. An even distribution of permeability was assumed across all surfaces, although the net airflow across party walls was assumed to be insignificant at normal operating pressures. Cracks were modeled at the top and bottom of external walls to account for differences in wind pressure according to the height of the building. Vented cellars and lofts were placed above and below the buildings, allowing free movement of outdoor air into these spaces. Cracks in the cellar ceilings and loft floors allowed air from the cellar and loft spaces, respectively, to enter the building based on the defined permeability of the envelope. In the case of flats, air from the cellar and loft entered the ground floor and top floor flats, respectively, and did not directly enter the studied mid-floor flat through these pathways. Internal walls, floors, and ceilings were also given cracks, allowing for the completion of the airflow network and the modeling of stack effects. Cracks were assigned reference air mass flow coefficients based on the building permeability and the surface area, and air mass flow exponents were set to 0.66, as per Jones et al. (2013). Windows and doors were modeled assuming two-way flow.

There are a number of studies that estimate indoor PM_2.5_ deposition and penetration into the building envelope. PM_2.5_ deposition was modeled using a deposition rate of 0.19/h (Long et al., 2001), with a penetration factor of 0.8 when windows were closed and 1.0 when windows were open. These values were used to perform an initial estimation of I/O ratios using a single-compartment box model (Long et al., 2001), typical air change rates of UK dwellings (BRE, 2009), and existing empirical studies of infiltration rates in the UK (Hoek et al., 2008), giving confidence that the values were suitable for modeling UK dwellings. Penetration factor and deposition rate of PM_2.5_ are also highly dependent on particle size (Long et al., 2001); however to simplify analysis, it was modeled as a single contaminant. Indoor pollutant levels and infiltration air change rates (ACH) were calculated every minute and output hourly.

#### Simulation of typical London dwellings

Two different scenarios were simulated to examine building performance and the influence of occupant behavior:

Scenario 1: No occupant interaction with ventilation components was modeled, and infiltration was only due to the permeability of the externally exposed façades of the dwellings. The dwellings were heated to a setpoint of 20°C, and internal gains due to occupant metabolism, hot water, and electrical equipment modeled as per Mavrogianni et al. (2012). Internal doors were assumed to be open at all times, with the exception of bedroom doors, which were closed at night. This represents the base-case performance of the building in terms of pollutant ingress.

Scenario 2: Temperature-driven window opening by building occupants. There are a number of both static and adaptive standards that can be used to estimate the temperature-related comfort of building occupants (CIBSE, 2013). The CIBSE Guide A summertime thermal comfort standards define an upper temperature threshold for comfort of 25°C for living rooms and 23°C for bedrooms (CIBSE, 2006). Internal temperatures were calculated inside the dwelling throughout the year. When internal operative temperatures exceeded the CIBSE summertime thermal comfort standards for living rooms during the day (07:00–22:00) or bedrooms during the night (22:00–07:00), windows were opened in the room. When internal temperatures dropped below the thresholds, they were closed. In both cases, the windows remained closed if the external temperature was greater than the internal temperature. Indoor heating and door-opening behavior was modeled as per Scenario 1. While there is a great deal of uncertainty when modeling building occupant window-opening behavior, the window opening assumptions used are broadly in line with existing field studies of occupant behavior (Dubrul, 1988; Fabi et al., 2012).

#### Data collation and analysis

Analysis of the hourly indoor PM_2.5_ pollutant predictions of the EnergyPlus models was carried out in SAS 9.3 (SAS Institute, 2013). Occupant exposure to indoor PM_2.5_ was considered to be dependent on the hourly room occupation schedule described in Shrubsole et al. (2012). A script was written to import the EnergyPlus output files and retrieve hourly PM_2.5_ levels from the room occupied at that point in time. The script then calculated the hourly I/O ratio, and then the monthly, hourly–monthly (the ratio for each time of the day, averaged across the month), seasonal, and yearly mean I/O ratio for the simulation period. The results for the typical dwellings were summarized according to the archetype and the occupation scenario. In addition, yearly average ACH values were calculated for the occupied rooms.

London experiences a significant diurnal and seasonal variation in outdoor PM_2.5_ levels. To account for this, the mean hourly–monthly outdoor PM_2.5_ level was obtained (London Air, 2014), and the percent deviation of the temporal values from the background mean calculated. These values were matched against the calculated mean hourly–monthly I/O ratios and used to calculate a temporally scaled I/O ratio for each month and season of the simulation period.

#### Sensitivity analysis

To explore the sensitivity of the model to variations in input parameters, a differential sensitivity analysis (DSA) was performed for penetration factor, deposition rate, building permeability, retrofit level, wind exposure, London climate, and occupant window and door-opening behavior. The methodology and results of the DSA are discussed further in the Appendix S1.

### GIS analysis

**Figure 3:**
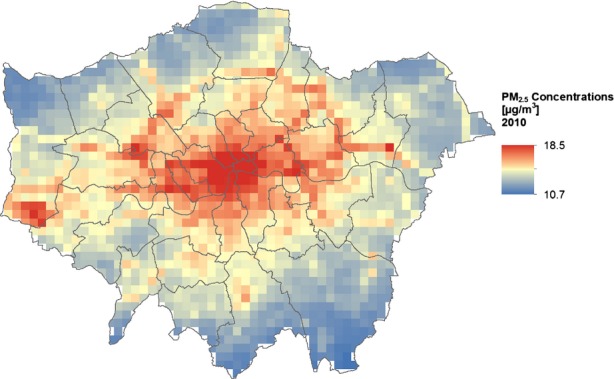
Estimated outdoor PM_2.5_ concentrations in Greater London for 2010 (DEFRA, 2011)

Geographical information systems (GIS) data were used to map the spatial variation in the I/O ratio of PM_2.5_ pollution based on the EnergyPlus results and to calculate the absolute indoor concentrations due to outdoor sources only based on predicted outdoor pollution levels. GIS analysis was performed in ArcGIS 10.1 (ESRI, 2013). Data obtained for the research area included the following:

- The GG Building Class topographic map, showing building footprints and building data, such as age and structure type (GG, 2013).
- Ordnance Survey (OS) Address Point data (OS, 2013), showing the number of domestic addresses within each building footprint.
- Department for Environment Food and Rural Affairs (DEFRA) map of estimated outdoor annual mean PM_2.5_ levels across London for 2010 (DEFRA, 2011) (Figure3).
- Postcode and borough boundary information from the UK Census (UK Data Service, 2013).

The GG Building Class database contains building footprint, built form, and age data for the Greater London Authority. Dwellings were filtered from the Building Class data to remove all non-domestic properties from the analysis. The Building Class database was filtered further to remove all dwellings that did not have built form or age information, or that did not match the archetypes used in this study. The remaining dwellings accounted for 76% of the known London domestic building stock (and 46% of the total domestic stock), or around 1.5 million dwellings.

The OS Address Point layer was used to determine the number of dwellings within a GG Building Class building footprint, identifying buildings with multiple occupancy. The Address Point layer was filtered to show only domestic addresses within the filtered Building Class footprints. The building classification data from the Building Class database were joined to the domestic address points through a spatial join.

The modeled I/O ratios for the different building archetypes for each scenario were joined to the Address Point database based on the building archetype classifications. The mean I/O ratios of the address points were calculated for each postcode area using the Spatial Overlay tool, and the results mapped to show differences in I/O ratio for dwellings in postcodes across London.

The I/O ratios of dwellings were then used to scale estimated outdoor concentrations of PM_2.5_ to predict absolute indoor concentrations. The DEFRA map for total annual mean outdoor PM_2.5_ concentrations from all sources in the GLA was joined spatially to the address point data. The local outdoor PM_2.5_ concentrations were then used to estimate average monthly indoor concentrations due to outdoor sources only for scenarios 1 and 2, based on the temporally scaled I/O concentration ratios for each month and season, with the assumption that the monthly variation in background PM_2.5_ levels was spatially consistent. The absolute indoor concentrations were summarized by calculating the mean monthly, seasonal, and yearly indoor concentration in each postcode.

## Results

### Building simulation

**Figure 4:**
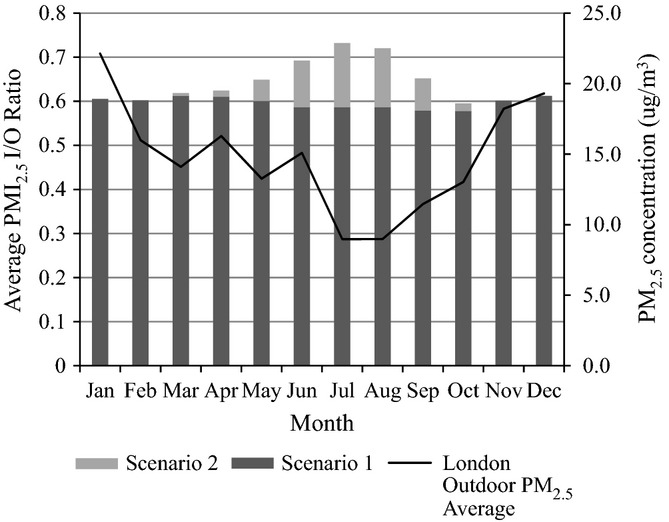
Average monthly PM_2.5_ I/O ratios for the bungalow living room under scenarios 1 and 2 (modeled with trickle vents), and monthly variation in London background PM_2.5_ levels (London Air, 2014)

An example of the monthly average I/O ratio for a bungalow with trickle vents can be seen in Figure4. The simulation results showed a slight decline in the PM_2.5_ I/O ratio in Scenario 1 during the summer period (May 1st–August 30th) relative to the winter period (Dec 1st–March 30th), largely attributable to a drop in infiltration caused by a 18% decrease in average wind speeds over this period. Compared with Scenario 1, Scenario 2 predicted an increase in average monthly I/O ratio during the summer period when windows were operable, exceeding winter levels. Sharp short-term increases in the indoor pollutant concentrations could be seen under Scenario 2, with window opening allowing the indoor PM_2.5_ levels to approach the simulated ambient outdoor levels when internal temperatures exceeded the 25°C threshold. Trickle vents were observed to increase the I/O ratios in all buildings.

**Figure 5:**
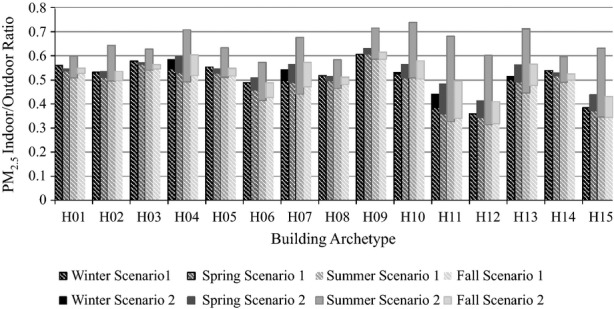
The seasonal average I/O ratio of PM_2.5_ pollution from outdoor sources, weighted according to room occupancy schedule and estimated frequency of trickle vents

The EnergyPlus results show a range of annual average I/O ratios of PM_2.5_ concentrations resulting from external sources in dwellings across London (Figure5). Detached and semi-detached properties with larger permeabilities showed higher amounts of pollution infiltration into the indoor air, while flats showed a much lower I/O ratio of pollution. Opening windows when temperatures exceed a comfort threshold led to an increase in the I/O ratio in all building archetypes, particularly in archetypes prone to overheating during the summer such as purpose-built flats (Mavrogianni et al., 2012). The yearly average ACH for the archetypes can be seen in Table S2. The results of the sensitivity analysis (Appendix S1) indicate that Scenario 1 I/O ratios are highly sensitive to permeability, penetration factor, and deposition rate, and less sensitive to weather file, retrofit level, and occupant window and door-opening behavior. Relative to Scenario 1, Scenario 2 results were more sensitive to retrofit level and less sensitive to permeability and penetration factor, reflecting the influence of temperature-coupled window opening. The degree of parameter sensitivity also varied between archetypes according to the number of exposed external walls, the tendency of buildings to overheat, and the cross-ventilation potential of the dwellings.

### GIS analysis

**Figure 6:**
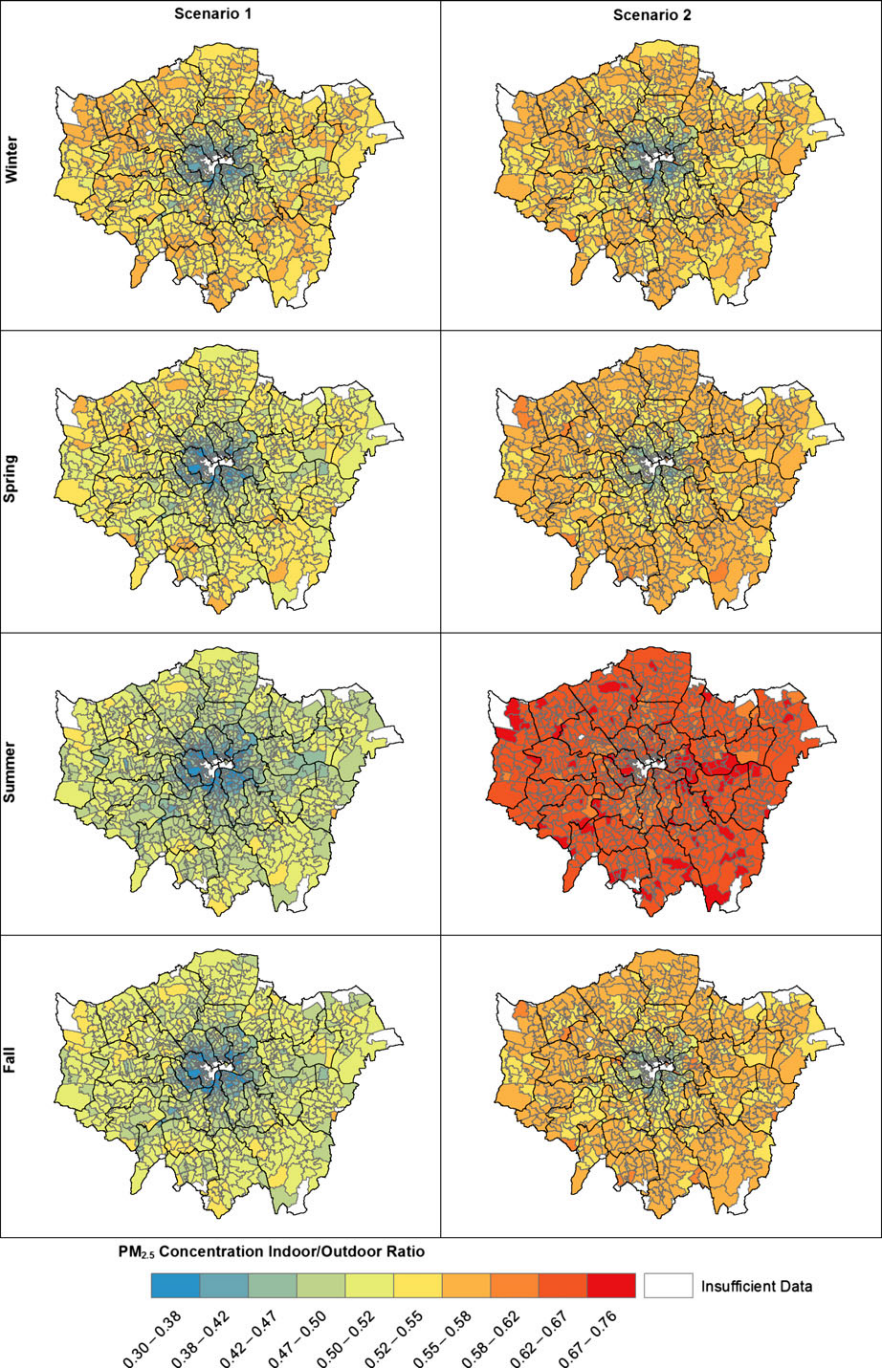
Seasonal average I/O PM_2.5_ ratios for dwellings across London for Scenario 1 and Scenario 2

The results of the GIS analysis indicate that many of the dwellings with a higher I/O PM_2.5_ ratio exist outside of Central London (Figure6). This is likely due to flats being the dominant dwelling type in the more densely populated center, while detached and semi-detached properties are more commonly found in the outskirts of the city. Interestingly, Figure6 contrasts with many outdoor pollution maps (for example, Figure3), which show elevated PM_2.5_ concentrations in Central London. There is insufficient building stock data to calculate average I/O ratios for 9.8% of postcodes in the research area. The majority of postcodes with insufficient data are located in Central London, where there are low numbers of residential properties. For Scenario 2, window opening during summer reduced much of the spatial variation seen in other seasons.

**Figure 7:**
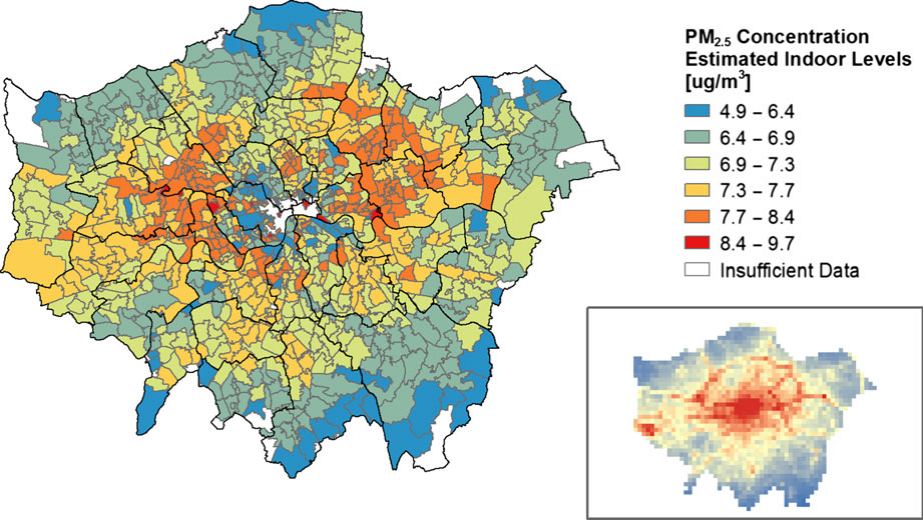
Estimated absolute indoor PM_2.5_ concentrations from outdoor sources, based on I/O ratio (Scenario 1) and estimated temporal and spatial variations in outdoor concentrations. The inset shows outdoor concentrations from Figure3

**Figure 8:**
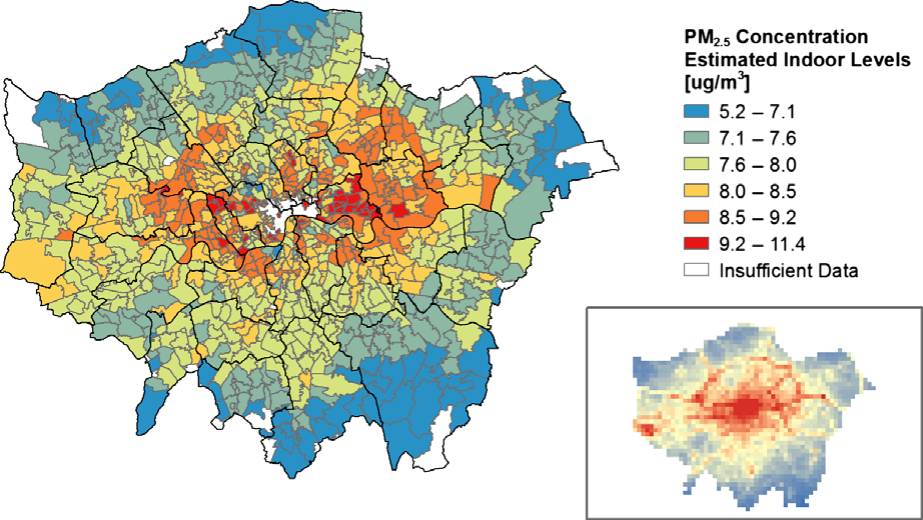
Estimated absolute indoor PM_2.5_ concentrations from outdoor sources, based on I/O ratio (Scenario 2) and estimated temporal and spatial variations in outdoor concentrations. The inset shows outdoor concentrations from Figure3

The results of the estimated indoor PM_2.5_ concentrations scaled for the DEFRA estimated levels of outdoor pollution can be seen in Figure7 (Scenario 1) and Figure8 (Scenario 2). Accounting for the modifying effect of buildings leads to an apparent inversion of the risk of PM_2.5_ exposure when compared to estimates of exposure based on outdoor concentration estimates. Locations with detached and semi-detached dwellings close to pollution sources, such as motorways, major roads, and mainline train tracks, become apparent as having high indoor PM_2.5_ levels from outdoor sources. Maps showing the seasonal variation estimated absolute levels can be seen in the Supplementary Materials (Figure S1). These results indicate that despite an increase in the infiltration due to window opening in Scenario 2 during the summer, the lower outdoor PM_2.5_ levels mean that the absolute indoor concentrations are still higher during the winter.

## Discussion

This work has shown how building simulation can be used to determine the indoor PM_2.5_ concentration from outdoor sources in a set of building archetypes, and the results mapped to estimate population exposure in indoor domestic environments. The differences in the PM_2.5_ I/O ratios predicted by EnergyPlus show, in some cases, a two-fold difference between dwelling types, indicating the importance of considering the potentially modifying effect of the building envelope when examining population exposure to air pollution. Occupant behavior can also have a major influence on exposure to outdoor pollution, with simulation results indicating that window opening during hot weather can cause spikes in indoor levels due to outdoor sources of PM_2.5_. Higher infiltration during the summer due to window opening is consistent with existing empirical studies (Hanninen et al., 2011). The results reflect the fact that dwellings with a higher exposed external surface area to internal volume ratio may be more susceptible to higher indoor concentration levels from outdoor sources.

The mapped results of the I/O ratios across London indicate that areas in outer London have higher numbers of detached and semi-detached dwellings that are more susceptible to outdoor pollution infiltration due to their greater externally exposed surface-area-to-volume ratio. This is in contrast to outdoor pollution data, which suggest that higher pollution levels can be generally found in Central London and near major roads and motorways. When PM_2.5_ I/O ratios are scaled against outdoor levels, there is an apparent inversion of exposure risk. The densely populated areas of Central London have the lowest estimated levels of indoor PM_2.5_ from outdoor sources despite the high outdoor concentrations due to attenuation by the predominant built form (flats and terraced dwellings) and their lower fabric permeability. The worst-affected areas were those around a busy circular road (North Circular) and along a major railway routes and highways heading East and West. This study has focused on London; however, the results may provide insight into other urban areas with dense modern flats in the city center and older detached properties in the suburbs or besides major traffic routes.

While there is a lack of empirical data for PM_2.5_ I/O ratios or infiltration rate in UK dwellings, the results are consistent with previous research. The measurements of PM_2.5_ in dwellings in Birmingham estimated an infiltration factor of 0.37 (Hoek et al., 2008), within the range of values obtained in the modeling work detailed. Indoor PM_2.5_ measurements obtained by Nasir and Colbeck (2013) are similar in magnitude, but are difficult to compare directly with modeled results without a schedule of indoor activities, an understanding of the outdoor levels during the measuring period, and building geometry and construction information. Other UK studies have found I/O ratios close to or greater than one due to the presence of indoor sources (Jones et al., 2000; Lai et al., 2006). International studies have found infiltration factors ranging from 0.30 to 0.70 in European studies (Hanninen et al., 2011), and 0.30 to 0.82 internationally (Chen and Zhao, 2011); these values are similar to the 0.33 to 0.60 (Scenario 1) and 0.45 to 0.62 (Scenario 2) ranges predicted by our model. The ACH of the archetypes (Tables S2) are similar to those in empirical studies of UK dwellings (AIVC, 1994; BRE, 2009; Dimitroulopoulou et al., 2005; Warren and Webb, 1980), while the lower ACH calculated for flats and attached dwellings relative to detached properties have been found in a number of previous studies (e.g. Persily et al., 2006).

There are a number of limitations that need to be considered in this work. While extensive, the coverage of the Building Class database lacked built form and/or age information for 32% of the dwellings in London, and not all of the known dwellings had a relevant archetype, which was modeled. Developing building archetypes for each combination of built form and age is unrealistic and would take a significant amount of time to simulate using currently available building simulation tools. The archetypes are intended to represent average buildings in London rather than a specific property, and deviations of individual buildings from the nominal archetypes are minimized when the results are considered over a wider geographical scale – in this case, postcode. Mid-floor flats are assumed to represent the majority dwelling type in multidwelling buildings and represent an ‘average’ of potential stack effects. It was not possible to model flats at different levels, as there is no information on the vertical distribution of addresses in the London building stock. The PM_2.5_ levels outside dwellings toward the top of the building are likely to be lower than levels at the bottom due to the generally larger distance from outdoor sources and the influence of local meteorology effects, specifically increases in wind speeds (Vardoulakis et al., 2008); however, higher wind pressures may increase infiltration rates. All simulations were run with wind speeds modified to reflect an urban terrain; however, terrain type in London varies from densely built central city areas to less dense outer suburbs. The increased exposure to wind forces in suburban areas is expected to lead to higher I/O ratios and potentially an increase in the apparent inversion of risk.

Internal sources are an important contributor to indoor PM_2.5_ levels, but have not been included in this study. While some building types allow higher levels of outdoor PM_2.5_ infiltration due to a high ACH, such buildings may also have a greater ability to allow indoor-produced PM_2.5_ to exfiltrate. This may mean that occupants of different building types may be exposed to different ratios of indoor-sourced to outdoor-sourced PM_2.5_. The chemical and toxicological profile of indoor sources of PM_2.5_ may differ from that of outdoor sources, meaning that they may lead to different health effects (Wilson et al., 2000).

Assumptions were also required in modeling the occupant behavior in Scenario 2. Window-opening behavior is complex, and indoor temperature is not the sole driver. Furthermore, top-level flats are more susceptible to overheating (Mavrogianni et al., 2012), a fact which suggests that occupants may open windows more frequently to reduce the internal temperatures and therefore temporarily drive up indoor levels of outdoor pollutants.

Only domestic properties were modeled and mapped in this study. While research suggests that people in the London spend over 80% of their time indoors (Kornartit et al., 2010), this includes time spent at work in, for example, offices, or engaging in leisure activities in shopping malls and theaters. Nonetheless, epidemiological analyses typically use the home postcode as an indicator of exposure, and this research is able to offer insight into how their dwellings may influence this exposure. This study has examined the indoor pollution levels throughout the day as an indicator of building performance and as such does not consider the fact that certain socio-demographic groups may spend a longer time than others in their home.

The modeling methodology used also carries with it a number of uncertainties. The EnergyPlus airflow network model is based on a validated airflow model, and initial comparisons between it and the indoor air quality model CONTAM give confidence in the results for contaminant transport (Taylor et al., 2013). Air leakage paths were assumed to be distributed across all bounding surfaces in the dwellings including party walls, which were assumed to be fully permeable. In reality, party walls may contribute up to 30% of air leakage at 50 Pa pressure differential in UK dwellings (Stephen, 2000). The calculated distribution of permeabilities for the EHS dwellings matched the measured distribution from the research of Stephen (2000) when the sheltering factor was included in calculations and was slightly higher when sheltering was excluded. Using the slightly higher values for buildings with party surfaces (equivalent to 22.5% higher for flats with three bounding surfaces) and applying them only to external walls, an attempt was made to compensate for the differences in permeability between external and party walls. However, further research is required to understand the permeability of different buildings and surface types in the UK housing stock. Modeling PM_2.5_ as a single contaminant is an important simplification and must be acknowledged.

Retrofit and airtightness measures, such as draught proofing, replacement windows, loft insulation, and the sealing of suspended floors, can reduce the permeability of a dwelling (Hong et al., 2004). There has been a significant focus on decarbonizing dwellings in the UK by limiting the heat loss through the building envelope. Building regulations specifying the air tightness requirements for new dwellings, as well as retrofits to reduce the permeability of existing structures, are one of the means to achieve energy use reductions. These measures will have the additional benefit of reducing pollutant infiltration into dwellings and reducing the I/O ratios of outdoor pollutants.

While a number of assumptions were necessary for this research, the results provide an insight into the potential modifying effects of the built form and building envelope on pollution infiltration in the London dwelling stock. Further field work is required to confirm the influence of built form and building permeability on the infiltration of outdoor pollution indoors. This research has implications for assessing the population exposure to pollutants from outdoor sources and can be used to supplement existing research into indoor air quality in London. Future research will increase the number of building archetypes to be representative of the entire UK, while additional pollutants will also be modeled from both outdoor and indoor sources.

## Conclusions

This analysis has mapped the potential indoor exposure of the London population to different PM_2.5_ levels from outdoor sources based on domestic building stock characteristics. The relative vulnerability of different dwellings to PM_2.5_ ingress has been demonstrated, and dwelling stock databases used to indicate areas where the stock is most vulnerable to high outdoor pollutant levels. This research indicates that flats have a reduced I/O ratio for PM_2.5_ from outdoor sources when compared to detached and semi-detached dwellings. The higher concentration of flats in Central London leads to an apparent inversion of exposure to indoor PM_2.5_ from outdoor sources when compared to estimates of exposure based on outdoor concentration estimates. The results can provide insight into other urban areas with spatial variations in building stock and outdoor pollution levels.

## Supporting Information

**Appendix S1.** Supporting information. **Table S1.** Thermal conductivity properties of the building envelope. **Table S2.** Modeled annual average Air Change Rates (h-1) or (ACH) for the dwelling archetypes under the two scenarios. **Table S3.** Variations in parameter inputs for DSA. **Table S4.** Variations in yearly average I/O ratio for Scenario 1 for different parameters. **Table S5.** Variations in summer average I/O ratio for Scenario 2 for different parameters. **Figure S1.** Seasonal changes in estimates absolute indoor and outdoor PM_2.5_ levels across London.
