# Supplementary material for: The modifying effect of the building envelope on population exposure to PM2.5 from outdoor sources
Source: Indoor Air. 2014 May 10;24(6):639–51. doi: 10.1111/ina.12116 (PMC4278446; doi:10.1111/ina.12116)
Supplement: Supplementary file 1 — Appendix S1. Supporting information. Table S1. Thermal conductivity properties of the building envelope. Table S2. Modeled annual average Air Change Rates (h-1) or (ACH) for the dwelling archetypes under the two scenarios. Table S3. Variations in parameter inputs for DSA. Table S4. Variations in yearly average I/O ratio for Scenario 1 for different parameters. Table S5. Variations in summer average I/O ratio for Scenario 2 for different parameters. Figure S1. Seasonal changes in estimates absolute indoor and outdoor PM2.5 levels across London. [file ina0024-0639-sd1.docx]

# Online supporting information for the following article published in Indoor Air

# DOI: 10.1111/ina.12116

**The modifying effect of the building envelope on population exposure to PM_2.5_ from outdoor sources**

Jonathon Taylor^a^, Clive Shrubsole^a^, Michael Davies^a^, Phillip Biddulph^a^, Payel Das^a^, Ian Hamilton^b^, Sotiris Vardoulakis^c^, Anna Mavrogianni^a^, Benjamin Jones ^d^, Eleni Oikonomou^b^

*^a^Bartlett School of Graduate Studies, UCL, London*

*^b^UCL Energy Institute, The Bartlett, UCL, London*

*^c^Centre for Radiation, Chemical and Environmental Hazards, Public Health England, Oxfordshire*

*^d^Department of Architecture and Built Environment, University of Nottingham, Nottingham*

Table S1. Thermal conductivity properties of the building envelope

| Archetype Code | U-Values | | | | |
| --- | --- | --- | --- | --- | --- |
|  | Walls | Ground Floors | Windows | Loft | Roof |
| H01 | 2.10 | 1.20 | 4.80 | 0.40 | 3.10 |
| H02 | 2.10 | 1.20 | 4.80 | 0.40 | 3.10 |
| H03 | 2.10 | 1.20 | 4.80 | 0.40 | 3.10 |
| H04 | 1.60 | 1.20 | 3.10 | 0.40 | 3.10 |
| H05 | 2.10 | 1.20 | 4.80 | 0.40 | 3.10 |
| H06 | 2.10 | 1.20 | 4.80 | 0.40 | 3.10 |
| H07 | 0.45 | 0.45 | 3.10 | 0.29 | 3.10 |
| H08 | 2.10 | 1.20 | 4.80 | 0.40 | 3.10 |
| H09 | 2.10 | 1.20 | 4.80 | - | 2.30 |
| H10 | 1.60 | 1.20 | 3.10 | 0.40 | 3.10 |
| H11 | 1.60 | 1.20 | 3.10 | - | 1.50 |
| H12 | 2.10 | 1.20 | 4.80 | - | 2.30 |
| H13 | 0.45 | 0.45 | 3.10 | 0.29 | 3.10 |
| H14 | 2.10 | 1.20 | 4.80 | 0.40 | 3.10 |
| H15 | 2.10 | 1.20 | 4.80 | - | 2.30 |

Table S2. Modelled annual average Air Change Rates (h^-1^) for the dwelling archetypes under the two scenarios.

|  | Scenario 1 | Scenario 2 |
| --- | --- | --- |
| H01 | 0.23 | 0.58 |
| H02 | 0.17 | 0.84 |
| H03 | 0.34 | 0.73 |
| H04 | 0.25 | 1.42 |
| H05 | 0.19 | 0.71 |
| H06 | 0.2 | 1.19 |
| H07 | 0.29 | 1.35 |
| H08 | 0.19 | 0.58 |
| H09 | 0.38 | 1.94 |
| H10 | 0.16 | 1.33 |
| H11 | 0.09 | 1.49 |
| H12 | 0.08 | 1.06 |
| H13 | 0.35 | 1.46 |
| H14 | 0.26 | 0.69 |
| H15 | 0.09 | 1.15 |
| Mean | 0.218 | 1.101333 |

*Differential Sensitivity Analysis*

A Differential Sensitivity Analysis (DSA) was performed to assess the sensitivity of the model to variations in model inputs. Each archetype was simulated at four orientations under Scenario 1 and 2 with a number of variations (Table S3). Parameter variations were selected based on the range of particle behaviours and building characteristics available in the literature, estimated ranges of occupant behaviour, and ranges in external environmental characteristics for London.

Table S3. Variations in parameter inputs for DSA.

| Category | Parameter Code | Parameter | Variation | Reference |
| --- | --- | --- | --- | --- |
| Particle behaviour | PB1 | Penetration factor | 0.6-1.0 | Chen and Zhao (2011) |
|  | PB2 | Deposition rate | 0.06-0.39h^-1^ | Meng et al., (2009); Ozkaynak et al. (1996) |
| Fabric Characteristics | FC1 | Retrofit level | No Retrofit – Full Retrofit | BRE (2009) |
|  | FC2 | Permeability | 3-30m^3^/h/m^2^ @50Pa | Stephens (2000) |
| Occupant Behaviour | OB1 | Window opening temperature threshold | 22-24 (Bedrooms)  24-26 (Living rooms) | - |
|  | OB2 | Internal doors open/closed | Always open/Always closed | - |
| External Environment | E1 | Wind exposure | Rural/City | - |
|  | E2 | Weather file | Gatwick, London Islington DSY | CIBSE (2013) |

Simulations were run for a year with 60 timesteps an hour and the results output hourly. For Scenario 1, the yearly average I/O ratio was considered. For Scenario 2, the average I/O ratio during the summer was examined, as this is when the internal temperatures were high enough to result in frequent window opening and significantly different I/O ratios than Scenario 1. The percentage difference in indoor PM_2.5_ concentrations from the baseline were calculated to demonstrate model sensitivity to each input variable, and the results added in quadrature to demonstrate the overall model uncertainty for the different scenarios.

The results of the DSA for Scenario 1 and 2 can be seen in Tables S4-S5. The results indicate a high level of sensitivity to the permeability of the building envelope (FC2), penetration factor (PB1) and deposition rate (PB2). The sensitivity of the predicted I/O ratios also varied with the different archetypes, with line-built flats most sensitive to variations in penetration factor and deposition rate. In Scenario 1, retrofit level and window-opening behaviour had a small influence on I/O ratio, while in Scenario 2 there was a greater sensitivity in dwellings with a tendency to overheat, such as flats. When window opening was enabled, the sensitivity of the model to penetration factor and building permeability is reduced.

Table S4. Variations in yearly average I/O ratio for Scenario 1 for different parameters.

|  | PB1 | PB2 | FC1 | FC2 | OB1 | OB2 | E1 | E2 | Quadrature Sum |
| --- | --- | --- | --- | --- | --- | --- | --- | --- | --- |
| H01 | 19.4% | 28.5% | 2.0% | 34.8% | 0.0% | 1.8% | 7.8% | 2.1% | 49.7% |
| H02 | 20.4% | 31.7% | 2.7% | 35.4% | 0.0% | 2.0% | 6.9% | 2.6% | 52.4% |
| H03 | 18.6% | 25.7% | 2.4% | 33.4% | 0.0% | 1.8% | 8.4% | 3.2% | 47.1% |
| H04 | 19.8% | 30.6% | 3.4% | 37.8% | 0.0% | 0.9% | 8.7% | 2.1% | 53.4% |
| H05 | 19.7% | 29.5% | 2.6% | 34.2% | 0.0% | 1.8% | 6.7% | 2.4% | 49.9% |
| H06 | 22.9% | 39.3% | 2.8% | 46.6% | 0.0% | 1.2% | 12.2% | 4.5% | 66.4% |
| H07 | 23.7% | 35.1% | 1.8% | 36.9% | 0.0% | 14.1% | 14.4% | 3.4% | 59.8% |
| H08 | 21.1% | 33.2% | 2.4% | 38.7% | 0.0% | 3.4% | 8.6% | 2.5% | 56.1% |
| H09 | 17.4% | 21.5% | 2.2% | 31.5% | 0.0% | 0.9% | 7.2% | 2.9% | 42.7% |
| H10 | 20.3% | 31.5% | 3.8% | 33.7% | 0.0% | 2.1% | 4.7% | 2.1% | 50.8% |
| H11 | 46.5% | 53.1% | 4.2% | 57.5% | 0.0% | 2.4% | 10.3% | 3.5% | 91.9% |
| H12 | 55.0% | 56.0% | 4.9% | 61.2% | 0.0% | 2.8% | 13.3% | 4.7% | 100.7% |
| H13 | 23.2% | 35.2% | 1.7% | 35.7% | 0.0% | 14.5% | 13.4% | 3.4% | 58.8% |
| H14 | 20.3% | 31.1% | 3.3% | 36.9% | 0.0% | 2.1% | 9.0% | 2.8% | 53.3% |
| H15 | 43.1% | 51.4% | 4.1% | 56.4% | 0.0% | 2.2% | 12.1% | 5.0% | 88.8% |

Table S5. Variations in summer average I/O ratio for Scenario 2 for different parameters.

|  | PB1 | PB2 | FC1 | FC2 | OB1 | OB2 | E1 | E2 | Quadrature Sum |
| --- | --- | --- | --- | --- | --- | --- | --- | --- | --- |
| H01 | 13.7% | 23.4% | 6.2% | 13.8% | 5.3% | 1.7% | 4.0% | 2.2% | 31.9% |
| H02 | 12.6% | 20.0% | 7.4% | 8.5% | 6.1% | 1.4% | 2.0% | 3.0% | 27.2% |
| H03 | 13.0% | 20.7% | 7.5% | 15.2% | 4.3% | 1.6% | 4.9% | 2.5% | 30.6% |
| H04 | 11.3% | 14.7% | 11.2% | 2.6% | 8.0% | 0.5% | 0.2% | 0.8% | 23.3% |
| H05 | 12.9% | 20.6% | 7.2% | 9.8% | 5.7% | 1.2% | 2.6% | 2.8% | 28.1% |
| H06 | 14.2% | 26.7% | 7.0% | 15.2% | 5.7% | 0.8% | 4.7% | 3.8% | 35.6% |
| H07 | 13.1% | 17.0% | 7.8% | 2.5% | 8.9% | 6.8% | 1.1% | 1.1% | 25.6% |
| H08 | 14.0% | 25.6% | 7.3% | 13.2% | 6.2% | 2.3% | 3.7% | 2.4% | 33.8% |
| H09 | 11.3% | 13.0% | 5.5% | 10.0% | 4.2% | 0.9% | 2.2% | 3.9% | 21.6% |
| H10 | 10.8% | 13.2% | 8.5% | 0.2% | 5.7% | 1.3% | 0.4% | 2.9% | 20.1% |
| H11 | 11.6% | 17.0% | 13.3% | 1.2% | 9.9% | 0.5% | 0.1% | 3.7% | 26.8% |
| H12 | 13.3% | 23.9% | 19.1% | 7.5% | 11.0% | 1.1% | 1.2% | 5.3% | 36.3% |
| H13 | 10.8% | 15.2% | 6.3% | 1.3% | 6.9% | 6.8% | 1.2% | 2.6% | 22.2% |
| H14 | 13.7% | 24.3% | 9.5% | 15.3% | 5.2% | 1.7% | 4.3% | 2.6% | 34.0% |
| H15 | 12.6% | 21.3% | 13.6% | 6.0% | 10.3% | 1.0% | 1.2% | 6.1% | 31.3% |

The values used to illustrate the model sensitivity to inputs are based on existing literature or best-estimates. In many cases, such as permeability, the values are extreme and unlikely to be observed within the modelled building stock. There are limitations to the DSA approach, including:

- DSA gives equal weights to the bounds of the parameter ranges. In reality, values near the mean are much more likely.
- DSA assumes the variables do not interact, and therefore will miss second-order effects due to interaction.

DSA was used rather than other techniques such as Monte Carlo Analysis (MCA) as there is little information on the distribution of the modelled parameters. Nonetheless, the sensitivity analysis provides an indication of the influence of input parameters on the calculated I/O ratios for the different archetypes.

**References**

BRE. (2009) *SAP: The Government’s Standard Assessment Procedure for Energy Rating of Dwellings*, Building Research Establishment, Watford, UK.

Chen, C., and Zhao, B. (2011) Review of relationship between indoor and outdoor particles: I/O ratio, infiltration factor and penetration factor, *Atmos Environ,* **45**, 275–288.

CIBSE (2013) *UK Weather Files*, Chartered Institution of Building Services Engineers, London, UK.

Meng, Q.Y., Spector, D., Colome, S., and Turpin, B. (2009) Determinants of indoor and personal exposure to PM2.5 of indoor and outdoor origin during the RIOPA study, *Atmos Environ*, 43, 5750-5758.

Ozkaynak, H, Xue, J., and Spengler, J. (1996) Personal Exposure to Airborne Particles and Metals: Results from the Particle TEAM Study in Riverside, California, *J Expo Anal Env Epid*, 6(1), 55-78.

Stephen, R. 2000. *Airtightness in UK Dwellings*, Building Research Establishment, Watford, UK.


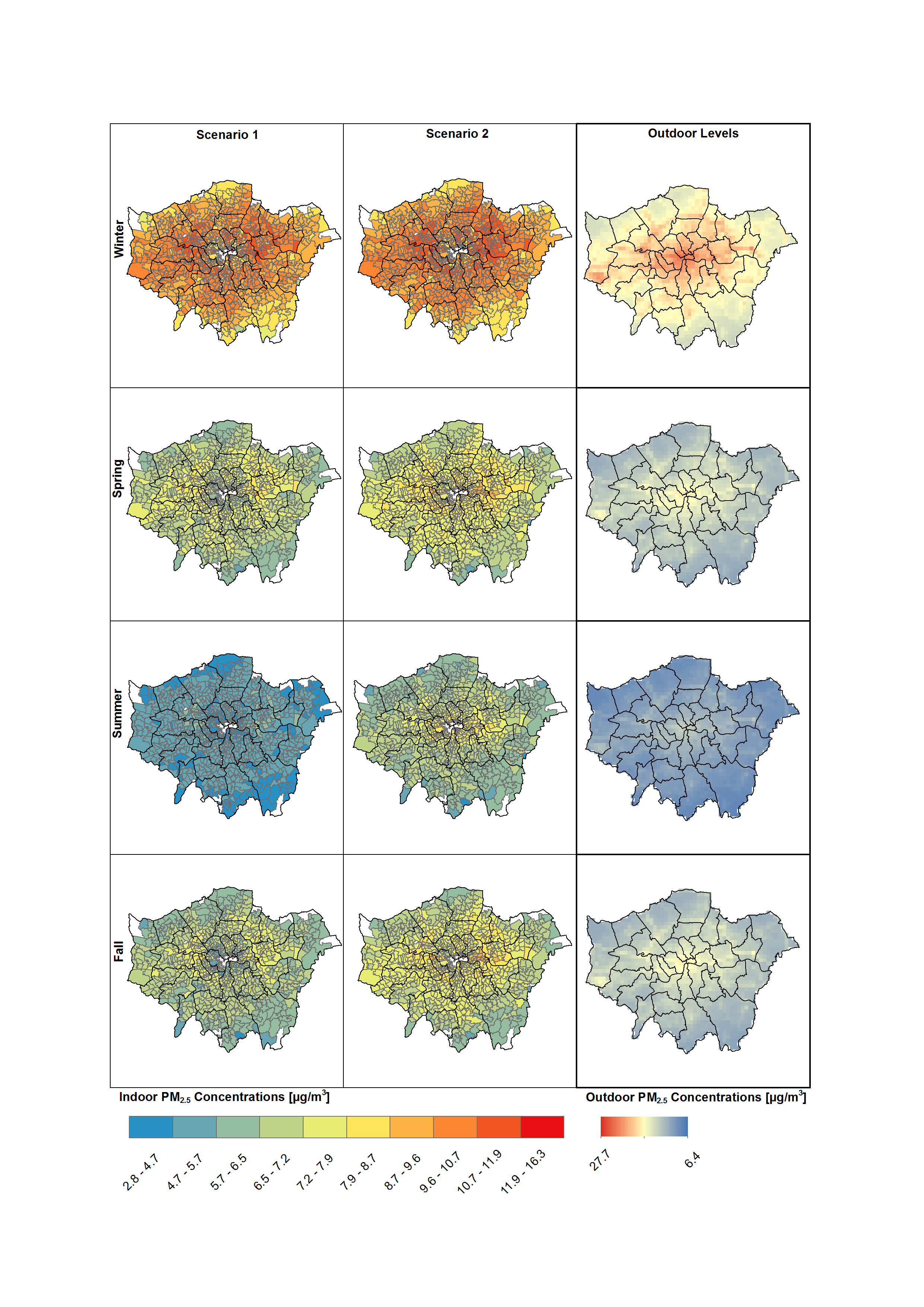


Figure S1. Seasonal changes in estimates absolute indoor and outdoor PM_2.5_ levels across London.
